# Inhibition of CDK4/6 as a novel therapeutic option for neuroblastoma

**DOI:** 10.1186/s12935-015-0224-y

**Published:** 2015-07-30

**Authors:** Ali Rihani, Jo Vandesompele, Frank Speleman, Tom Van Maerken

**Affiliations:** Center for Medical Genetics, Ghent University, De Pintelaan 185, 9000 Ghent, Belgium

**Keywords:** Cell cycle, Neuroblastoma, Palbociclib, Cyclin D1, Targeted therapy

## Abstract

**Background:**

Neuroblastoma is a neural crest-derived tumor and is the most common cancer in children less than 1 year of age. We hypothesized that aberrations in genes that control the cell cycle could play an important role in the pathogenesis of neuroblastoma and could provide a tractable therapeutic target.

**Methods:**

In this study, we screened 131 genes involved in cell cycle regulation at different levels by analyzing the effect of siRNA-mediated gene silencing on the proliferation of neuroblastoma cells.

**Results:**

Marked reductions in neuroblastoma cellular proliferation were recorded after knockdown of *CCND1* or *PLK1*. We next showed that pharmacological inhibition of cyclin D1 dependent kinases 4/6 (CDK4/6) with PD 0332991 (palbociclib) reduced the growth of neuroblastoma cell lines, induced G1 cell cycle arrest, and inhibited the cyclin D1-Rb pathway.

**Conclusion:**

Selective inhibition of CDK4/6 using palbociclib may provide a new therapeutic option for treating neuroblastoma.

**Electronic supplementary material:**

The online version of this article (doi:10.1186/s12935-015-0224-y) contains supplementary material, which is available to authorized users.

## Background

Neuroblastoma is a solid childhood tumor that arises from the sympathoadrenal lineage of the neural crest during development [1]. It represents the most common cancer in children younger than 1 year [2] and is a heterogeneous disease that may either have a very good prognosis (e.g., stage 4S neuroblastoma) or a dismal outlook (e.g., *MYCN*-amplified neuroblastoma) [1]. Neural crest-derived malignancies such as neuroblastoma are believed to have defects in the regulatory circuits that are essential for embryonic development and regulation of cellular differentiation, in particular at the G1-S transition [1, 3]. The involvement of cell cycle regulators such as *PLK1* [4], *TRIM16* [5], *WEE1* [6], *CDK4/6* [7], and *CCND1* [8] in various aspects of neuroblastoma pathogenesis suggests that deregulation of the normal cell cycle could be an important factor in driving neuroblastoma tumorigenesis and opens the possibility that more oncogenic cell cycle regulators could be identified by screening a large set of cell cycle regulators.

In this study, we used an siRNA library targeting 131 cell cycle regulators that belong to different gene families involved in cell cycle regulation. The siRNA library was used to assess the effect of knockdown of cell cycle regulators on the proliferation of neuroblastoma cells. This approach is expected to result in the identification of genes that are required for proliferation or survival of neuroblastoma cells, and therefore may serve as new therapeutic targets. Our screen showed that several cell cycle regulators are critical for neuroblastoma cellular proliferation. The strongest reductions in cellular proliferation were observed after knockdown of *CCND1* or *PLK1*. To translate these findings into a pharmacologically useful approach, we explored the potential therapeutic utility of palbociclib, a small-molecule inhibitor of cyclin D1 associated kinases, mainly CDK4 and CDK6. We showed that palbociclib, which is widely used in clinical trials, inhibits the growth of neuroblastoma cells in vitro. Moreover, cell cycle analysis demonstrated that palbociclib induces cell cycle arrest exclusively in G1. In addition, we showed that inhibiting CDK4/6 by palbociclib suppresses the cyclin D1-pRb pathway by inhibiting the phosphorylation of Rb and the expression of E2F target genes. Our data suggest that the CDK4/CDK6 inhibitor palbociclib may offer therapeutic benefit for the treatment of neuroblastoma.

## Results

### Screening of neuroblastoma cellular proliferation using an siRNA library of 131 cell cycle regulators

**Fig. 1:**
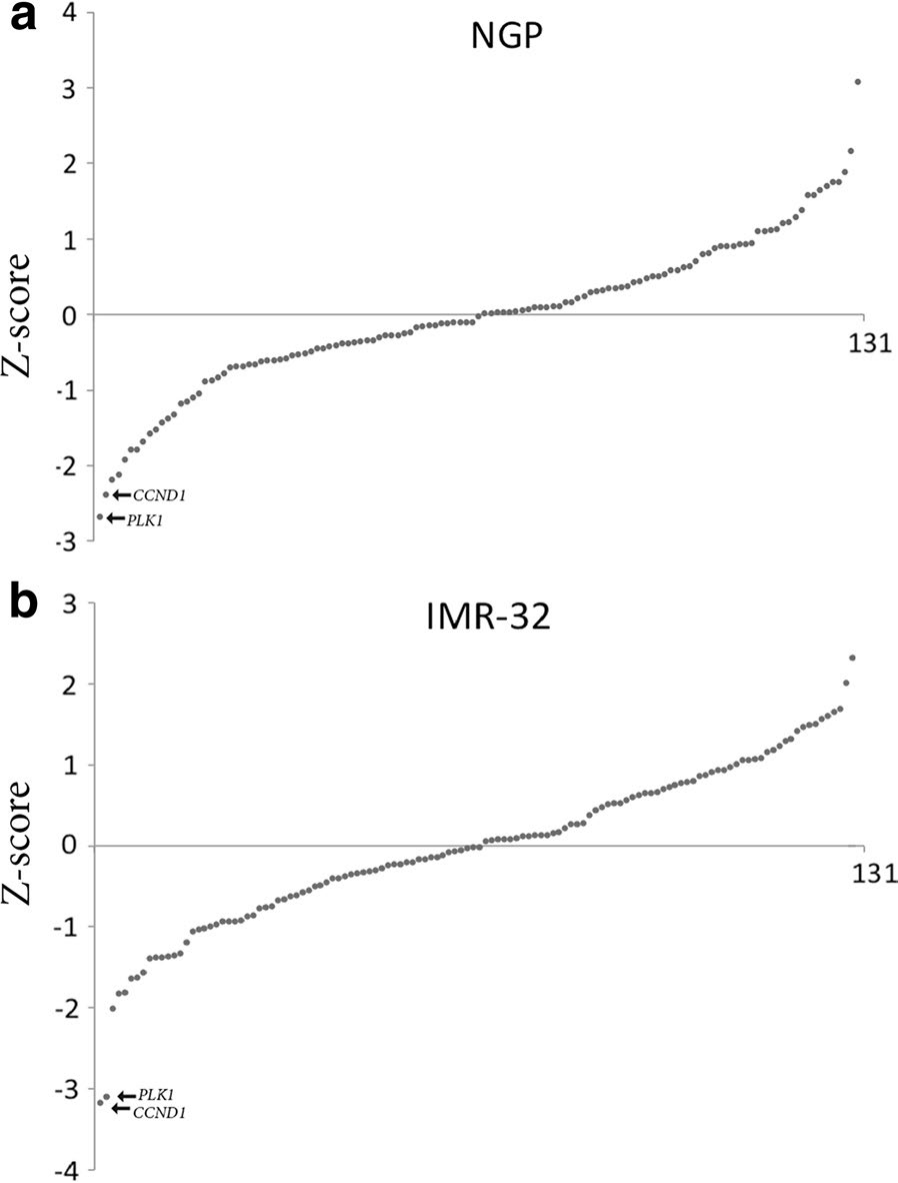
siRNA library screening of cell cycle regulators. NGP cells (**a**) and IMR-32 cells (**b**) were transfected with the siRNA library including negative controls (scrambled siRNA) and their growth was assessed using the xCELLigence system. The data shown in the *graphs* represent the cellular proliferation measurements recorded at 48 h post transfection. The data are depicted as Z score values (Additional file 1: Table S1), which represent the number of standard deviations from the average for every measurement. Positive Z score values indicate an increase in cell growth, whereas *negative values* represent a decrease in cell growth.

We used an siRNA library to target 131 cell cycle regulators in two neuroblastoma cell lines (NGP and IMR-32) followed by monitoring of neuroblastoma cell growth in real time. The siRNA library included siRNAs that target genes which belong to several gene families involved in cell cycle regulation at different levels, such as cyclin dependent kinases (CDKs), members of the retinoblastoma protein family, DNA replication factors like the cell division cycle proteins (CDCs), members of the CIP/KIP family, and the INK4 family of cell cycle inhibitors. Our results showed that several genes of these families are required for the growth of both NGP and IMR-32 cells. In particular, silencing of *CCND1* and *PLK1* showed the most pronounced effects in reducing neuroblastoma cellular proliferation (Fig. 1; Additional file 1: Table S1).

### Knockdown of *CCND1* in neuroblastoma cell lines

**Fig. 2:**
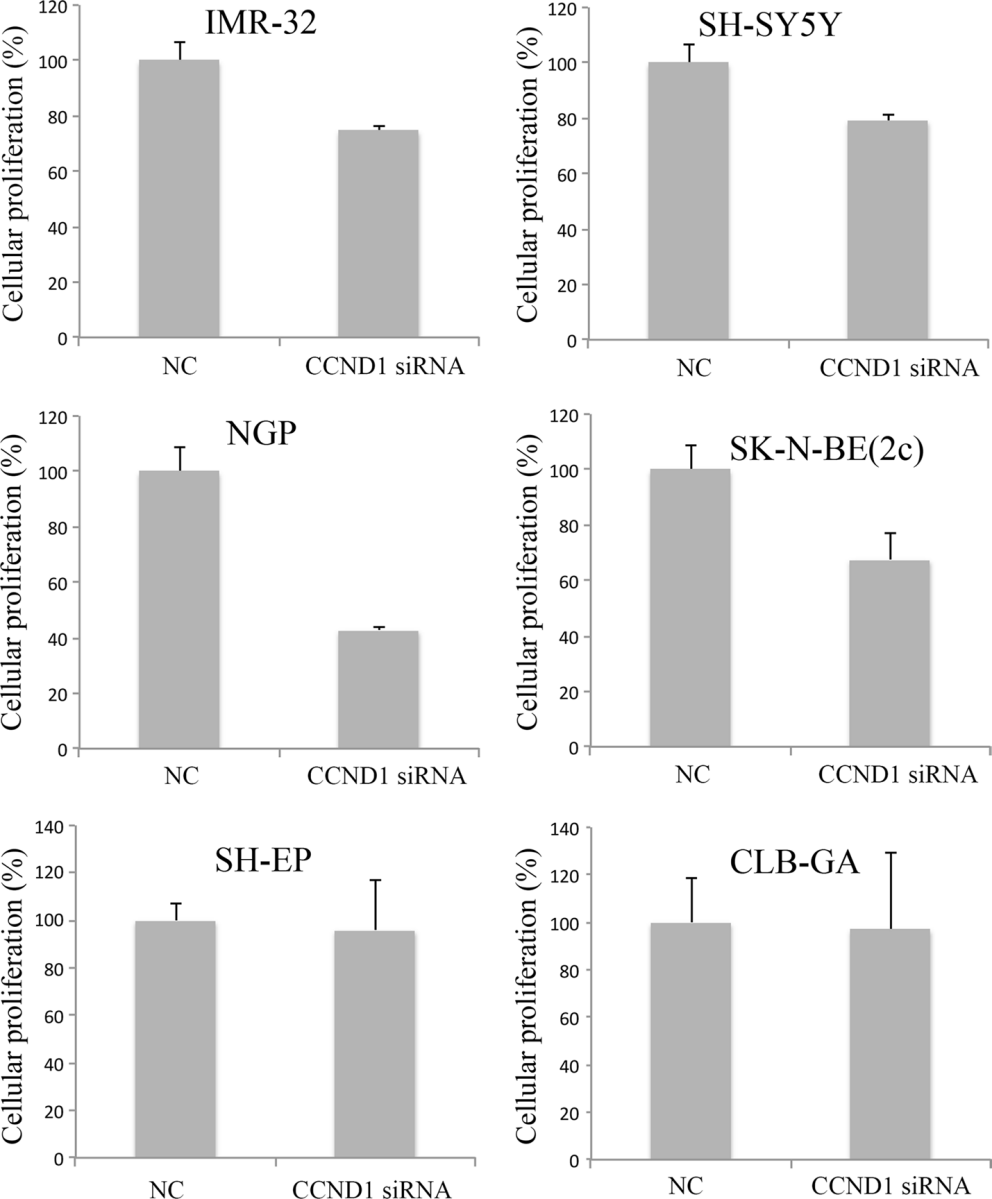
Assessment of cellular proliferation after knockdown of *CCND1*. The cellular proliferation was measured in real time using the xCELLigence system. The data shown represents the percentage of cellular proliferation after 72 h of knockdown of *CCND1*. *NC* scrambled siRNA negative control. *Error bars* represent the standard deviation (n = 2).

*CCND1* and *PLK1* were the primary hits of our siRNA library screen. As inhibition of PLK1 using small molecule compounds has already been extensively studied in neuroblastoma [4, 9], we decided to select *CCND1* for further experiments. We first measured the mRNA expression levels of *CCND1* in a panel of 31 neuroblastoma cell lines (Additional file 1: Figure S1) and evaluated the effects of *CCND1* knockdown on the proliferation of eight neuroblastoma cell lines in real time using the xCELLigence system. The cell lines had various levels of *CCND1* expression and were either *MYCN* amplified or *MYCN* nonamplified and either mutant or wild-type for *TP53* (Additional file 1: Table S2). Our results showed that knockdown of *CCND1* induces a reduction in cellular proliferation in the majority of the neuroblastoma cell lines in comparison to the scrambled siRNA negative control (Fig. 2). The real-time data is shown in Additional file 1: Figure S2.

### Targeting the cyclin D1-CDK4/CDK6 complex with palbociclib

**Fig. 3:**
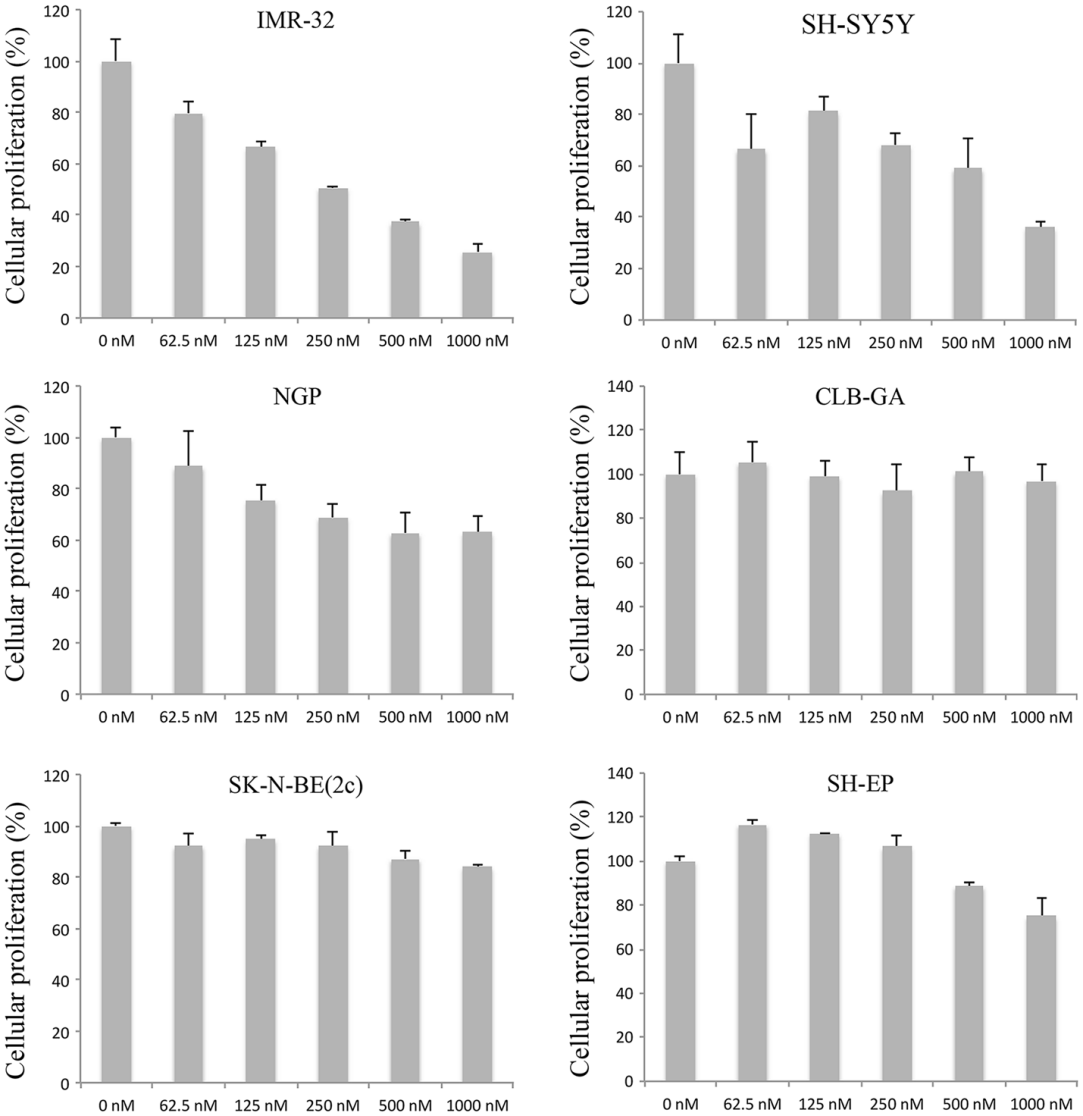
Assessment of cellular proliferation after treatment with palbociclib. The cellular proliferation data was measured in real time using the xCELLigence system. The data shown represents the percentage of cellular proliferation 48 h after treatment with palbociclib. *Error bars* represent the standard deviation (n = 2).

Targeting cyclin D1 with small molecule compounds is currently not possible. However, an alternative approach to inhibit its activity is by inhibiting the associated kinases, CDK4 and CDK6. Indeed, a small molecule compound known as PD 0332991 or palbociclib has been reported to be a potent and highly selective inhibitor of CDK4 and CDK6 [10]. We treated neuroblastoma cells with this compound and evaluated the effect on cellular proliferation using xCELLigence. Our results showed that palbociclib inhibits the growth of IMR-32, SH-SY5Y, and NGP cells in a time- and dose-dependent manner. In contrast, other neuroblastoma cell lines such as SK-N-SH and CLB-GA were relatively resistant to the treatment. Other cell lines such as SH-EP responded at relatively high concentrations (Fig. 3). IC50 values are shown in Table 1 and the real-time data is shown in Additional file 1: Figure S3. The effect of palbociclib on the proliferation of neuroblastoma cells is very similar to the effect induced by the knockdown of *CCND1*, which suggests that both treatments induce their effects by targeting the same pathway.

**Table 1:** IC50 values of neuroblastoma cell lines treated with palbociclib

Cell line	IC50 (nM)
IMR-32	261
SH-SY5Y	676
NGP	2077
SH-EP	2211
SK-N-SH	7,482 × 10^5^
NLF	1,109 × 10^4^
CLB-GA	2,579 × 10^5^
SK-N-BE(2c)	1,285 × 10^4^

### Palbociclib induces G1 arrest in neuroblastoma cells

**Fig. 4:**
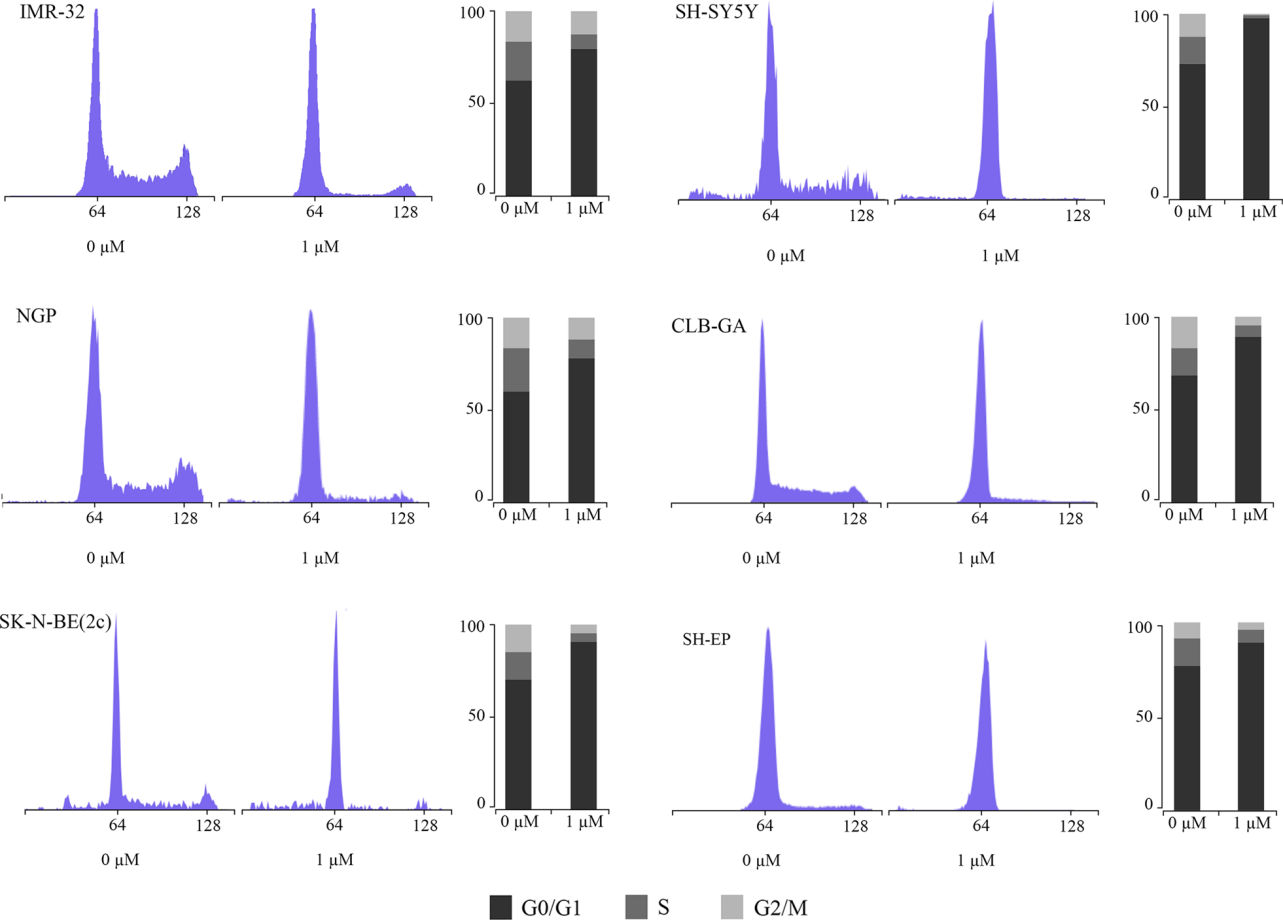
Cell cycle analysis after treatment with palbociclib. DNA histograms generated by flow cytometry for neuroblastoma cell lines treated with palbociclib for 24 h. The DNA histograms show the distribution of cell populations in each phase of the cell cycle. The percentages of cells are shown to the *right* of each histogram.

We evaluated the effect of palbociclib on the progression of the cell cycle in the neuroblastoma cell lines. The cells were exposed to vehicle control (0 µM) or 1 µM of palbociclib for 24 h. Cell cycle analysis showed that palbociclib induces an increase in the percentage of cells in G1 and a decrease in the other phases of the cell cycle in the neuroblastoma cell lines (Fig. 4). This induction of a G1 cell cycle arrest is consistent with what is expected after selective inhibition of CDK4 and CDK6 [10].

### Palbociclib inhibits Rb phosphorylation and the Rb/E2F pathway in neuroblastoma cells

**Fig. 5:**
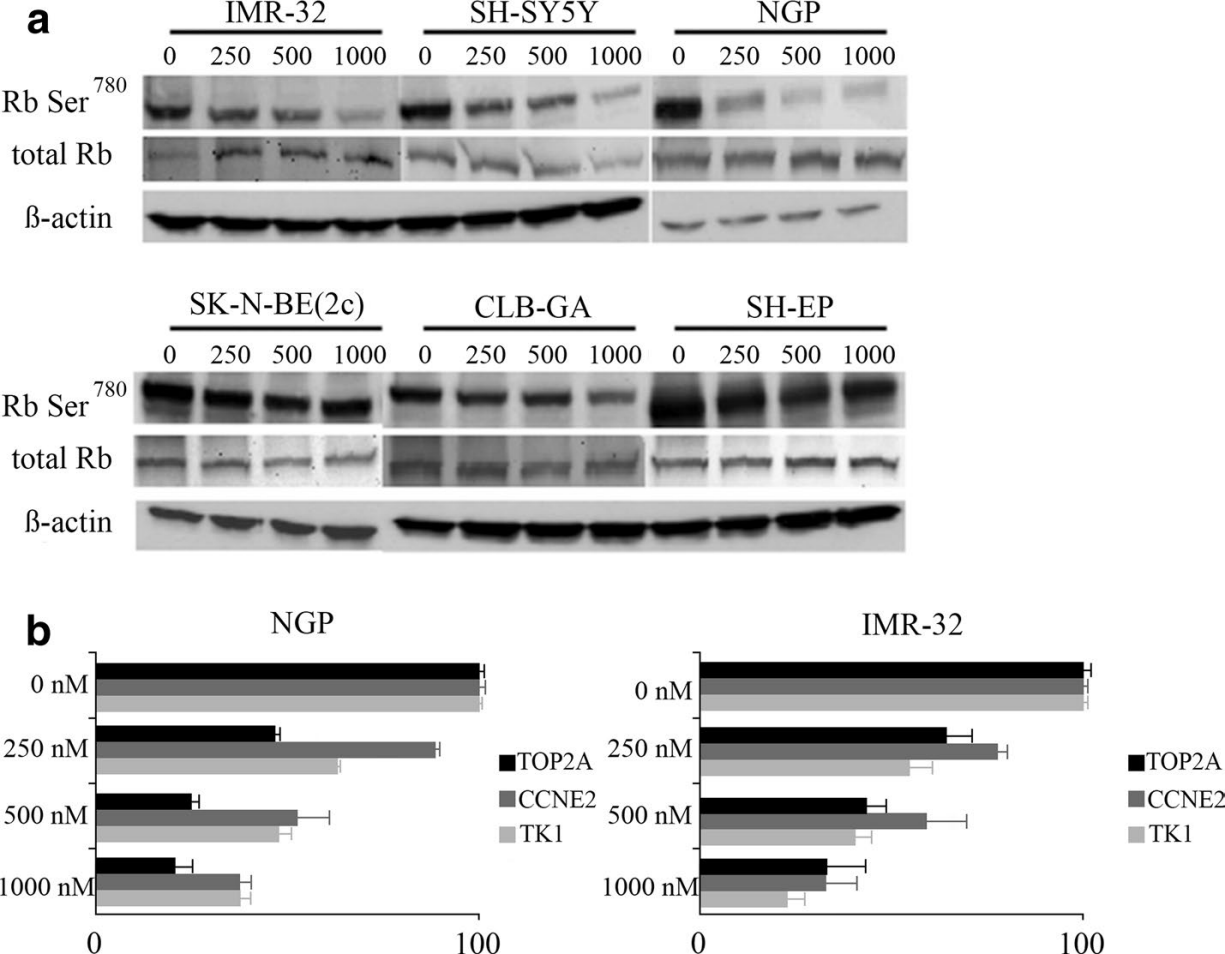
Palbociclib inhibits Rb Ser^780^ phosphorylation and reduces the expression of E2F target genes. Neuroblastoma cell lines were treated with different concentrations of palbociclib for 24 h. Western blot analysis was performed using anti-Rb Ser^780^ to detect specific phosphorylation of Rb at Ser^780^. Anti-Rb was used to detect total Rb, and anti-β actin as a loading control (**a**). RT-qPCR data of NGP and IMR-32 cells showing the expression levels of E2F target genes after 24 h of treatment with palbociclib. Error bars represent the standard error of the mean (n = 2) (**b**).

The cyclin D1-CDK4/6 complex specifically phosphorylates Rb at Ser^780^ [11]. Reduction in Rb phosphorylation at Ser^780^ is indicative of CDK4 and CDK6 inhibition. Upon treatment of neuroblastoma cells with 0, 250, 500, or 1,000 nM of palbociclib, we evaluated the degree of Rb phosphorylation by Western blot analysis. Treatment with palbociclib inhibited the phosphorylation of Rb in a dose-dependent manner only in the cells that showed a decrease in cellular proliferation after palbociclib treatment (Fig. 5a). Phosphorylation of Rb by the cyclin D1-CDK4/6 complex releases E2F from the inhibitory grip of Rb [12]. Therefore, hypophosphorylation of Rb due to inhibition of CDK4/6 should lead to the inhibition of E2F activity. We evaluated the effect of palbociclib on the Rb/E2F pathway in neuroblastoma cells by measuring the mRNA expression levels of direct E2F target genes, topoisomerase 2A (*TOP2A*), cyclin E2 (*CCNE2*), and thymidine kinase (*TK1*). IMR-32 and NGP cells were treated with 0, 250, 500, or 1,000 nM of palbociclib for 24 h. Treatment with palbociclib reduced the expression of *TOP2A*, *CCNE2*, and *TK1* in a dose-dependent manner, as expected (Fig. 5b).

## Discussion

Several cell cycle regulators, such as *PLK1* [4], *TRIM16* [5], *WEE1* [2, 6], *CDK4/6* [1, 7], and *CCND1* [1, 3, 8], have been shown to be involved in the tumorigenesis of neuroblastoma. This suggests that neuroblastoma tumors might be addicted to individual activated oncogenes in the process of cell cycle regulation and that additional oncogenic cell cycle regulators could play a role in neuroblastoma tumorigenesis. We screened two neuroblastoma cell lines using a commercially available arrayed siRNA library representing 131 cell cycle regulators from different gene families to identify genes that are required for the proliferation or survival of neuroblastoma cells and that might serve as new therapeutic targets. The gene families included cyclin dependent kinases (CDKs), members of the retinoblastoma protein family, DNA replication factors such as the cell division cycle proteins (CDCs), members of the CIP/KIP family, and the INK4 family of cell cycle inhibitors. Real-time monitoring of cell growth showed that knockdown of *CCND1* and *PLK1* had the strongest effects in reducing the proliferation of neuroblastoma cells. *PLK1* has previously been extensively studied in neuroblastoma and has been shown to inhibit the transactivation activity of p53 and to promote cell survival [4, 13, 14]. Moreover, targeting PLK1 with small molecule compounds induced apoptosis and growth arrest in neuroblastoma tumor-initiating cells [5, 9] and reduced the growth of neuroblastoma xenografts in nude mice [4, 6]. Cyclin D1 has also been studied to some extent in neuroblastoma and has previously been shown to be highly expressed in a subset of neuroblastoma cell lines and tumors due to gene amplification or GATA3 binding [7, 8, 15, 16]. Cyclin D1 belongs to a group of proteins known as cyclins, which are involved in the temporal coordination of each mitotic event during the cell cycle. Cyclins exhibit a cell-cycle dependent pattern of expression and degradation, and they activate the CDKs. Cyclin-CDK complexes phosphorylate target proteins such as Rb and coordinate the progression into the next phase of the cell cycle [8, 12]. Cyclin D1 activates CDK4 and CDK6 and this initiates the phosphorylation of the tumor suppressor protein Rb resulting in the activation of E2F. The activity of E2F leads to the transcriptional activation of E2F-responsive genes, such as *TOP2A*, *CCNE2*, and *TK1*, that are essential for DNA synthesis and cell cycle progression [4, 9, 10, 17]. Despite being an attractive therapeutic target, small molecule compounds that can directly and specifically inhibit cyclin D1 do not exist at present. It is possible, however, to inhibit cyclin D1 indirectly by several means. For example, inhibition of *CCND1* can be achieved by mTOR inhibitors, since *CCND1* mRNA translation is mTOR-dependent [10, 18], or by inhibitors of glycogen synthase kinase 3 β (GSK3β), which phosphorylates cyclin D1 at Thr-286 suggested to regulate the turnover and the intracellular distribution of cyclin D1 [10, 19]. The most effective approach to inhibit cyclin D1 activity is by inhibiting its associated kinases CDK4 and CDK6 [11, 12]. Pan-CDK inhibitors can inhibit CDKs but induce also considerable toxicity due to off-target effects [12, 20]. In contrast, early phase clinical trials have shown that specific and potent inhibition of CDK4/6 by small molecule drugs, such as palbociclib [4, 21–24] and R547 [5, 25], can result in antitumor activity with tolerable side effects. Recently, it has been shown that inhibition of CDK4/6 by LEE011 reduces the growth of neuroblastoma tumors with *MYCN* amplification in murine xenograft models [7]. In this study, we used palbociclib, a selective CDK4/6 inhibitor that has already shown promising antitumor activity in several clinical trials in other cancer types [12]. Treatment of neuroblastoma cells in vitro with palbociclib resulted in variable effects: some cell lines, such as IMR-32, SH-SY5Y, and NGP, were sensitive, whereas other cell lines, such as SK-N-SH and CLB-GA, were relatively resistant. It is worth mentioning that NGP cells have *CDK4* amplification. Additional studies are needed to explain the different sensitivity to this compound.

## Conclusions

We show that palbociclib induces a G1 cell cycle arrest in neuroblastoma cells, which is in line with previous reports in other cancer types [10]. Palbociclib also induces hypophosphorylation of Rb at Ser^780^, indicative of CDK4/6 inhibition, in those neuroblastoma cell lines that show a reduction in cellular proliferation after treatment. Upon hypophosphorylation, Rb forms a complex with E2F and we show that treatment with palbociclib reduces the expression levels of E2F target genes involved in cell cycle progression, such as *TOP2A*, *CCNE2*, and *TK1*. Taken together, our results suggest that inhibition of CDK4/6 may provide an effective strategy to treat a subset of neuroblastoma tumors.

## Methods

### Cell lines and palbociclib treatment

Neuroblastoma cell lines were grown as monolayer cultures at 37°C and 5% CO_2_ in a humid atmosphere. The culture medium was RPMI 1640 (GIBCO, Life Technologies) containing 10% Fetal Calf Serum (FCS), 2 mmol/l glutamine, and the following antibiotics: Penicillin (1%), Kanamycin (1%), and Streptomycin (1%). Palbociclib (Selleck Chemicals) was dissolved in DMSO and stored as a 10 mmol/l stock solution at −20°C. Keeping the final concentration of DMSO constant, cells were treated with palbociclib ranging from 0 to 2 µmol/l for the time periods indicated.

### siRNA transfection

Neuroblastoma cells were seeded in RPMI 1640 (GIBCO, Life Technologies) with 10% FCS and without antibiotics and transfected with the siRNA library against 131 cell cycle regulators (Dharmacon Cell Cycle ON-TARGET*plus*, Thermo Scientific) at a concentration of 100 nM using Dharmafect 2 transfection reagent (Thermo Scientific). The genes targeted by the siRNA library are listed in Additional file 1: Table S3. Transfections with siRNA against CCND1 and scrambled negative control siRNA were done using smart pools of siRNAs (Dharmacon, Thermo Scientific).

### Cell growth assessment

xCELLigence MP (Roche Diagnostics) was used to monitor cell proliferation in real time. Background impedance was measured before seeding the cells using 40 µl of RPMI 1640 containing 10% FCS and always subtracted as blank value. 1 × 10^4^ cells in 50 µl of RPMI containing 10% FCS were added. Cell proliferation was measured with a programmed signal detection every 1 h and the signal was normalized to the transfection or palbociclib treatment time point where the cell index at every time point was divided by the cell index at the time of transfection. Data acquisition and analysis was performed with the RTCA software (version 1.2, Roche Diagnostics). IC50 values were defined as the drug concentration that inhibited cell proliferation by 50% at 48 h of treatment and were calculated using GraphPad Prism (version 6).

### RNA extraction and RT-qPCR (reverse transcription-quantitative polymerase chain reaction)

Analysis of gene expression was performed by RT-qPCR according to MIQE guidelines [26]. RNA extraction, cDNA synthesis and RT-qPCR were performed as described previously [27]. The primers were designed using an in-house developed web tool (http://www.primerXL.org) and the sequences can be found in Additional file 1: Table S4. *HMBS*, *HPRT1*, *SDHA*, *TBP*, and *YWHAZ* were used for normalization. Biogazelle’s *qbase* + qPCR data-analysis software version 3.0 [28] was used to quantify the relative expression of the genes.

### Western blot

Neuroblastoma cells treated with palbociclib (Selleck Chemicals) for 24 h were harvested and washed using ice-cold PBS, centrifuged and the supernatant discarded. The pellet was solubilized in RIPA lysis buffer (Pierce) containing protease and phosphatase inhibitor mixture (Roche). Cell lysates were placed on ice for 30 min and centrifuged for 10 min at 12,000 rpm at 4°C. The protein concentration was measured using the Bio-Rad Protein Assay (Bio-Rad). Protein samples were mixed at 1:1 ratio with Laemmli denaturation buffer (Bio-Rad) and β-mercaptoethanol (Sigma Aldrich) at a final dilution of 1/40 and boiled for 10 min at 95°C. Approximately 25 µg of protein was loaded and fractionated using a 10% SDS-PAGE gel (Bio-Rad). The protein was transferred onto a nitrocellulose membrane (Bio-Rad) and immunoblotted with rabbit monoclonal antibody against phospho-Rb (Ser^780^) (Cell Signaling Technology), rabbit polyclonal antibody against total Rb (Abcam), and mouse monoclonal antibody against β-actin (Sigma Aldrich) as a loading control. Secondary antibody was anti-mouse HRP-linked (Cell Signaling Technology) and anti-rabbit HRP-linked (Cell Signaling Technology). Visualization of the proteins was done using ChemiDoc-It^2^ Imaging System (UVP, Sopachem).

### Cell cycle analysis

Neuroblastoma cells were treated with palbociclib for 24 h, harvested, washed with PBS and resuspended in 100 µl of PBS. 100 µl of DNA Prep lysis buffer (Beckman Coulter) was added to the cells and the suspension was vortexed and incubated for 5 min. The DNA was stained using 2 ml DNA Prep stain (Propidium iodide) and the suspension was placed in the dark for 30 min at room temperature. Cellular DNA content was analyzed using the S3 cell sorter (Bio-Rad).

## Additional file

Additional file 1. Real-time viability data, expression data and primers sequences.

## Abbreviations

CDK4/6: cyclin D1 dependent kinases 4/6
CDCs: cell division cycle proteins
*TOP2A*: topoisomerase 2A
*CCNE2*: cyclin E2
*TK1*: thymidine kinase
GSK3β: glycogen synthase kinase 3 β
FCS: fetal calf serum
RT-qPCR: reverse transcription-quantitative polymerase chain reaction
