# Supplementary material for: Inhibition of CDK4/6 as a novel therapeutic option for neuroblastoma
Source: Cancer Cell Int. 2015 Jul 30;15:76. doi: 10.1186/s12935-015-0224-y (PMC4518532; doi:10.1186/s12935-015-0224-y)
Supplement: Additional file 1. — Real-time viability data, expression data and primers sequences. [file 12935_2015_224_MOESM1_ESM.docx]

**Supplementary Data**

**
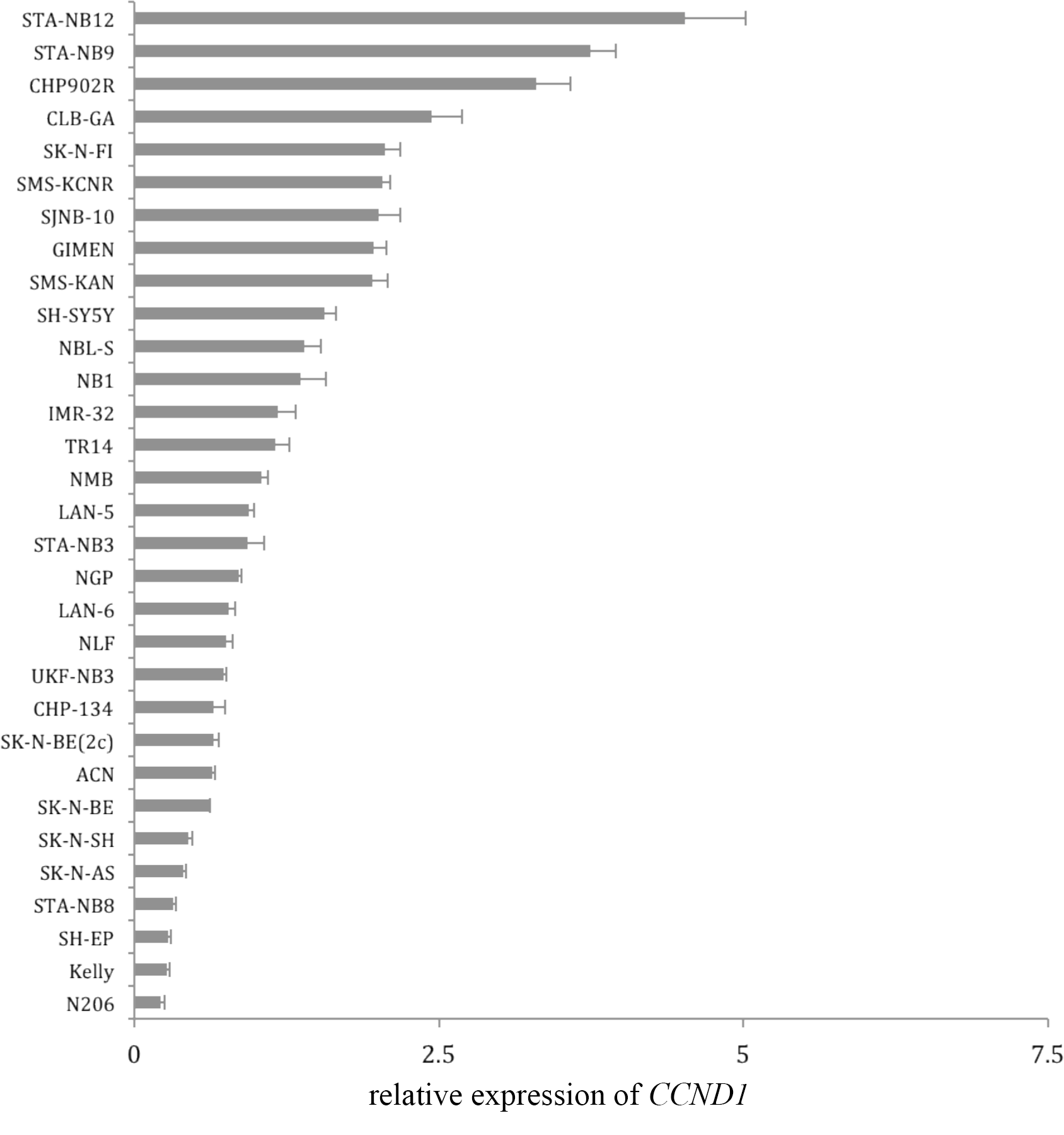
**

**Figure S1:** ***CCND1* mRNA expression levels**. *CCND1* expression in 31 neuroblastoma cell lines was evaluated using RT-qPCR. Error bars represent the standard error of the mean (n=2)


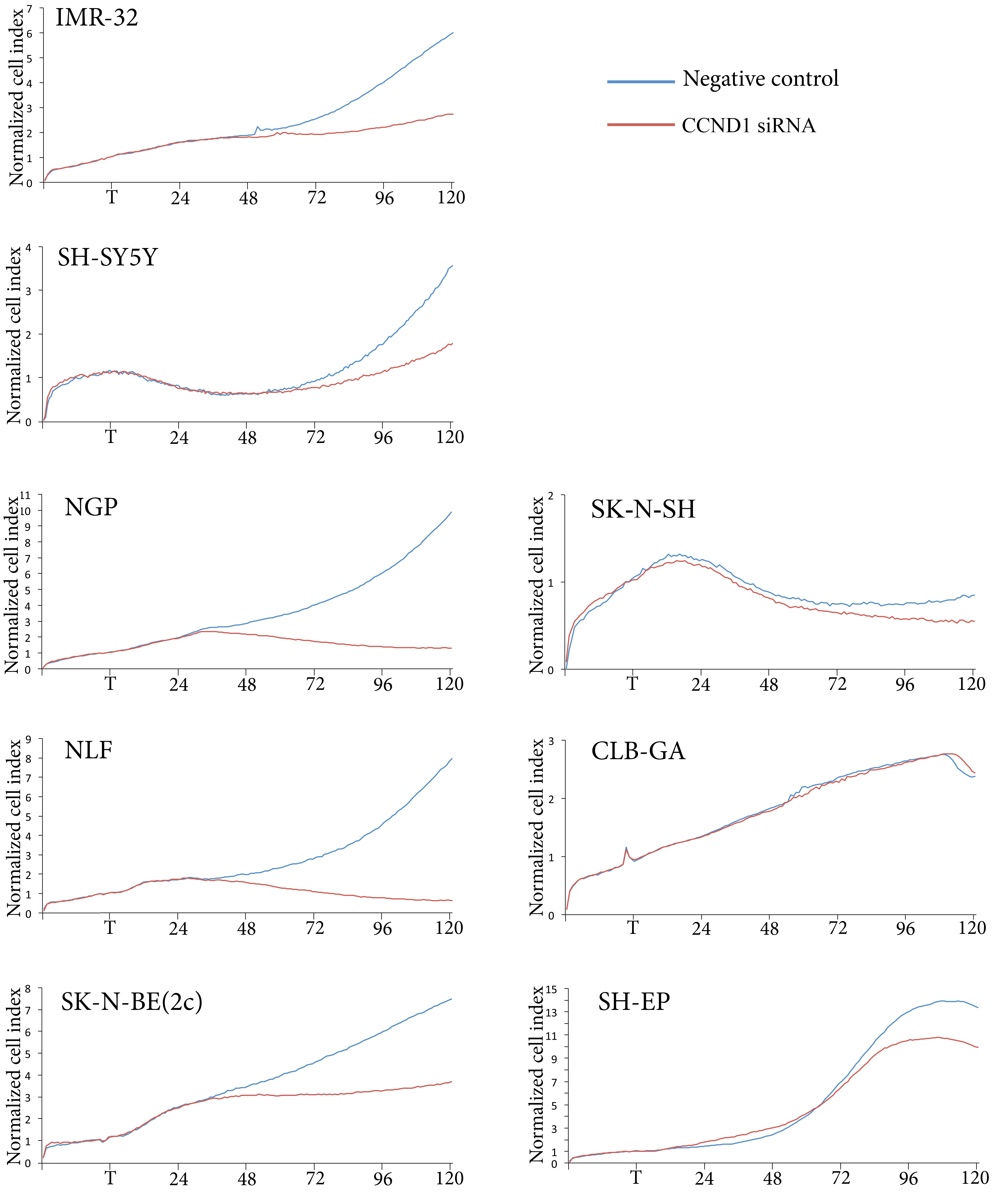


**Figure S2**: **Assessment of cell viability after knockdown of *CCND1*.** Normalized cell index of the cell viability data measured in real time using the xCELLigence system up to 120 hours post transfection with siRNA against *CCND1* or scrambled siRNA negative control. The x-axis represents the time in hours. T, time of transfection.

**
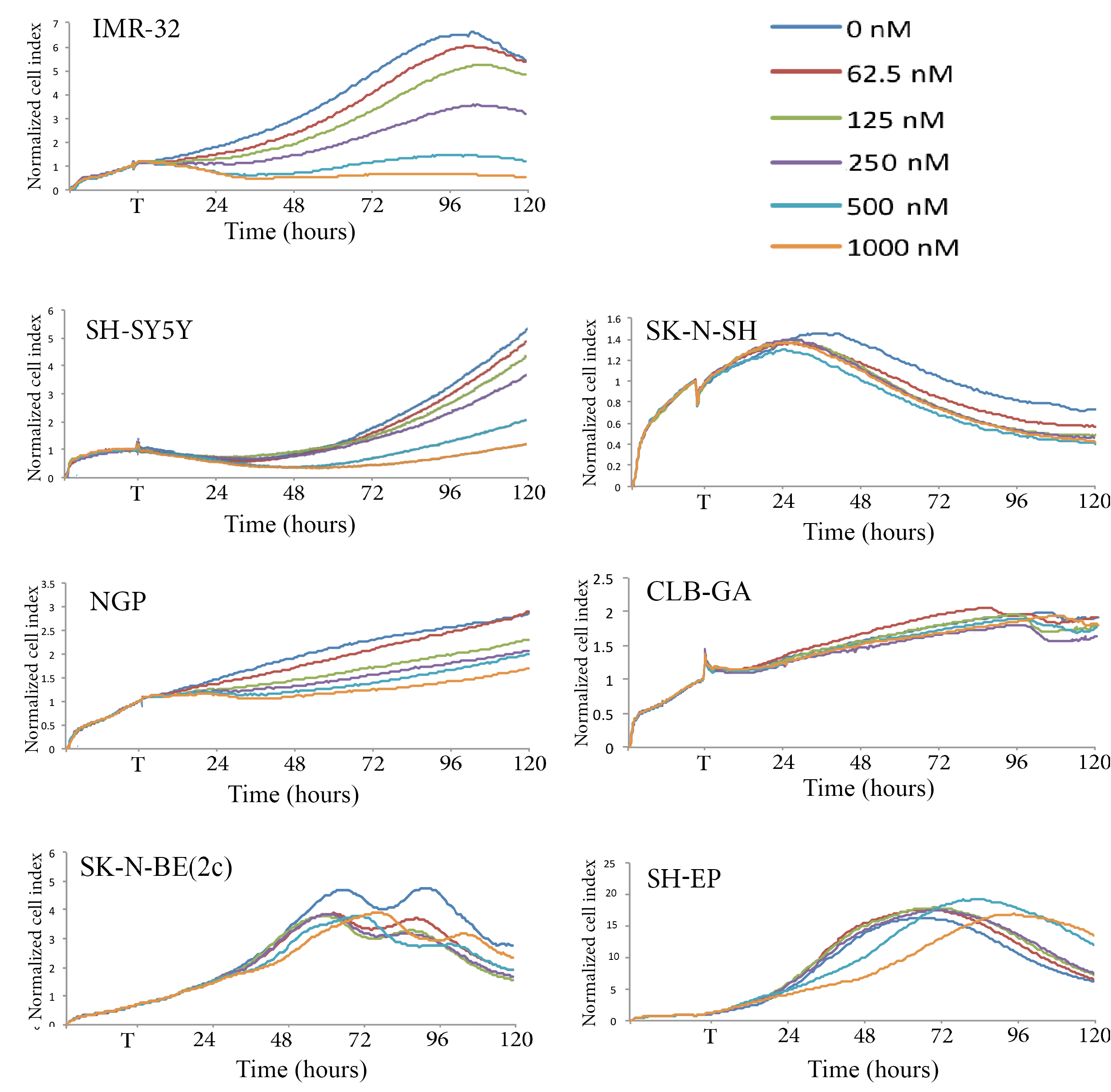
**

**Figure S3: Assessment of cell viability after treatment with palbociclib.** Normalized cell index of the cell viability data measured in real time using the xCELLigence system after treatment with a dilution series of palbociclib. The x-axis represents the time in hours. T, start of treatment.

**Table S1: Z-score values representing the assessment of the cell viability**

| **IMR-32** | | **NGP** | |
| --- | --- | --- | --- |
| **Gene** | **Z-score** | **Gene** | **Z-score** |
| *CCND1* | -3.172686386 | *PLK1* | -2.671637405 |
| *PLK1* | -3.09553892 | *CCND1* | -2.385689766 |
| *CCNL1* | -2.008635243 | *CENPC1* | -2.181816869 |
| *CENPE* | -1.816671459 | *RAD17* | -2.123544738 |
| *RAD51* | -1.807492405 | *GMNN* | -1.921153861 |
| *RAD17* | -1.634337135 | *CCND2* | -1.788493503 |
| *CENPC1* | -1.618814392 | *RAD51* | -1.784766102 |
| *E2F1* | -1.563598467 | *CENPE* | -1.682308463 |
| *CDKN2A* | -1.386776039 | *CCND3* | -1.571436747 |
| *CHEK1* | -1.381517379 | *CDKN2A* | -1.518102251 |
| *ORC3L* | -1.37950485 | *E2F2* | -1.427386931 |
| *MDM2* | -1.355818516 | *CENPB* | -1.375713648 |
| *HUS1* | -1.328273451 | *CDKN3* | -1.317150593 |
| *CCNI* | -1.195095105 | *CDC37* | -1.173886271 |
| *CENPH* | -1.051536491 | *CDKN2D* | -1.150647791 |
| *CDKN2C* | -1.027872523 | *RBP1* | -1.094805035 |
| *RBP1* | -1.016042136 | *PIN1* | -1.042864146 |
| *CENPB* | -0.991719238 | *MDM2* | -0.887041478 |
| *CKS2* | -0.934531316 | *CDK4* | -0.868033569 |
| *CDC34* | -0.932552815 | *CDK10* | -0.826827608 |
| *AURKB* | -0.931870887 | *CCNH* | -0.774594698 |
| *CDC16* | -0.916186542 | *CCNF* | -0.699147163 |
| *ANAPC2* | -0.867087724 | *CDK5R2* | -0.689033126 |
| *CDKN1C* | -0.861567414 | *RBL1* | -0.685479818 |
| *CENPF* | -0.773484866 | *CCNG2* | -0.662003761 |
| *CDC25C* | -0.758661167 | *CDC16* | -0.655619739 |
| *CDK5R1* | -0.745704535 | *CDK2* | -0.623699627 |
| *CCND2* | -0.672738236 | *CDKN2C* | -0.606355873 |
| *PTGIS* | -0.656663274 | *CCNE1* | -0.602806464 |
| *ATR* | -0.627730986 | *YWHAZ* | -0.587024699 |
| *CDK10* | -0.614092426 | *HUS1* | -0.578796788 |
| *CENPA* | -0.56773981 | *TP73L* | -0.546712367 |
| *CDC27* | -0.546581551 | *CDK5R1* | -0.530841122 |
| *CRI1* | -0.503978563 | *CCNI* | -0.514010518 |
| *CCNT1* | -0.483844172 | *ORC6L* | -0.493996241 |
| *GMNN* | -0.446133308 | *CDKN1C* | -0.447891137 |
| *TFDP1* | -0.404281299 | *TFDP1* | -0.442830589 |
| *CDKN2D* | -0.400120037 | *E2F6* | -0.424925233 |
| *ORC1L* | -0.376511115 | *PTGIR* | -0.413371577 |
| *CDKN3* | -0.350162771 | *CDK5* | -0.379365685 |
| *MCM6* | -0.336489378 | *E2F4* | -0.37554854 |
| *CHEK2* | -0.330442797 | *CCNL1* | -0.363115447 |
| *RAD9A* | -0.308719193 | *ORC4L* | -0.359880213 |
| *CCNH* | -0.305179029 | *TFDP2* | -0.347476419 |
| *ORC6L* | -0.272781306 | *GAK* | -0.335358209 |
| *PTGIR* | -0.23684342 | *CHEK1* | -0.296316173 |
| *CCNA1* | -0.223347666 | *MCM6* | -0.281581261 |
| *CCND3* | -0.223347666 | *RB1* | -0.273053652 |
| *RBL2* | -0.200905534 | *CCNT1* | -0.270256941 |
| *FOS* | -0.199633641 | *ANAPC2* | -0.254587069 |
| *RBL1* | -0.169868268 | *MYC* | -0.231966083 |
| *PLK3* | -0.164795021 | *PLK2* | -0.164520451 |
| *MCM8* | -0.144548394 | *RBL2* | -0.151341419 |
| *PIN1* | -0.143731624 | *ORC5L* | -0.14281381 |
| *CCNA2* | -0.116284965 | *CDC25C* | -0.136192474 |
| *E2F6* | -0.078027138 | *RAD9A* | -0.113354798 |
| *CCNF* | -0.061730723 | *TP73* | -0.111804324 |
| *TP73* | -0.053886908 | *RPA3* | -0.097074818 |
| *CDC2* | -0.03513553 | *CDC2* | -0.025342634 |
| *CDKN2B* | -0.023468545 | *CDT1* | 0.010278642 |
| *CCNG2* | -0.016041545 | *HIPK2* | 0.018316709 |
| *ATM* | 0.062380178 | *CCNG1* | 0.026890275 |
| *RAD52* | 0.069445383 | *ORC3L* | 0.028513601 |
| *SKP1A* | 0.078429855 | *JARID1A* | 0.029288838 |
| *CDKS1B* | 0.079075317 | *CDK6* | 0.037837727 |
| *YWHAZ* | 0.089864637 | *RAD1* | 0.052545952 |
| *CDK2* | 0.123071773 | *E2F5* | 0.074583173 |
| *ORC4L* | 0.124168983 | *CDC20* | 0.091310864 |
| *HIPK2* | 0.12837525 | *CDC34* | 0.093051961 |
| *PLK2* | 0.136420535 | *CENPF* | 0.098697372 |
| *CDC37* | 0.136710333 | *CDK9* | 0.104438848 |
| *ORC5L* | 0.153572708 | *ATR* | 0.111623662 |
| *CDC45L* | 0.16194167 | *CDC6* | 0.16269584 |
| *CDT1* | 0.221059125 | *CDC25A* | 0.168499496 |
| *CDKN1B* | 0.261156404 | *CDC25B* | 0.213187652 |
| *CDK4* | 0.271732083 | *CDKN2B* | 0.24223427 |
| *CDK5R2* | 0.275617718 | *CCNE2* | 0.295599575 |
| *TFDP2* | 0.376550957 | *PTGIS* | 0.307598976 |
| *RPA3* | 0.433724867 | *CKS2* | 0.324911523 |
| *E2F2* | 0.471502787 | *SKP2* | 0.341709411 |
| *AURKA* | 0.511318031 | *CDC7* | 0.342609194 |
| *CCNT2* | 0.524727381 | *RAD52* | 0.363416051 |
| *GAK* | 0.52869071 | *CENPA* | 0.376584807 |
| *RB1* | 0.561957779 | *CCNA2* | 0.421538923 |
| *MNAT1* | 0.601979516 | *CDC27* | 0.438369527 |
| *CDC6* | 0.627016585 | *CDKN1A* | 0.477634783 |
| *SKP2* | 0.650985725 | *CCNC* | 0.503950847 |
| *CDC25B* | 0.653611778 | *AURKA* | 0.513036822 |
| *MCMDC1* | 0.664870817 | *MKI67* | 0.537069173 |
| *JARID1A* | 0.695091313 | *TP53* | 0.58435864 |
| *E2F3* | 0.718659787 | *CDC45L* | 0.585782404 |
| *CDKL5* | 0.745610417 | *CCNT2* | 0.622925806 |
| *CDK5* | 0.772949183 | *E2F3* | 0.64528588 |
| *MCM7* | 0.789836649 | *PLK3* | 0.708442116 |
| *CCNE1* | 0.802954016 | *CRI1* | 0.793415959 |
| *CDC25A* | 0.863645611 | *CDK8* | 0.817530158 |
| *RAD1* | 0.878047825 | *E2F1* | 0.873796622 |
| *E2F4* | 0.907971532 | *CCNB3* | 0.89917986 |
| *CCNG1* | 0.929110702 | *AURKB* | 0.904983516 |
| *MYC* | 0.940122356 | *SKP1A* | 0.907632533 |
| *TP73L* | 0.966259 | *CDK3* | 0.927037411 |
| *E2F5* | 1.012487391 | *CDK7* | 0.933507972 |
| *PCNA* | 1.052836635 | *CHEK2* | 0.942694334 |
| *CDC7* | 1.059358955 | *FOS* | 1.098862479 |
| *CCNE2* | 1.068905947 | *ORC1L* | 1.107643718 |
| *CDK6* | 1.088080622 | *CDKL1* | 1.116086907 |
| *CDK9* | 1.155128531 | *CENPH* | 1.131014744 |
| *ORC2L* | 1.179436007 | *CDKL5* | 1.215988588 |
| *CCNC* | 1.235296386 | *PCNA* | 1.220828343 |
| *CCNB1* | 1.295987981 | *MNAT1* | 1.28982445 |
| *CDK3* | 1.32053739 | *CDKN1B* | 1.38249139 |
| *CCNB2* | 1.413279601 | *CDKL2* | 1.576553277 |
| *CDK8* | 1.462760116 | *MCM7* | 1.582864094 |
| *CDC20* | 1.496474821 | *ORC2L* | 1.650309726 |
| *CDKL2* | 1.502857396 | *CCNA1* | 1.697763005 |
| *CDKL1* | 1.566618643 | *MCMDC1* | 1.749540082 |
| *CCNB3* | 1.602855593 | *ATM* | 1.751156646 |
| *CDKN1A* | 1.650757196 | *CCNB2* | 1.880578188 |
| *MKI67* | 1.694001197 | *MCM8* | 2.165842433 |
| *CDK7* | 2.009003379 | *CCNB1* | 3.076131444 |

**Table S2**: Mutational status of *TP53* and copy number status of *MYCN*.

|  | *MYCN* | *p53* |  |
| --- | --- | --- | --- |
| IMR-32 | A | wt |  |
| SK-N-SH | NA | wt |  |
| NGP | A | wt |  |
| SHEP | NA | wt |  |
| SH-SY5Y | NA | wt |  |
| SK-N-BE(2c) | A | mut |  |
| NLF | A | mut |  |
| CLB-GA | NA | wt |  |

A, amplified; NA, nonamplified; wt, wild-type; mut, mutant.

**Table S3:** Dharmacon Cell Cycle ON-TARGET*plus*

| **Symbol** | **GeneID** | **Accession** |
| --- | --- | --- |
| *APC2* | 29882 | NM_013366 |
| *ATM* | 472 | NM_000051 |
| *ATR* | 545 | NM_001184 |
| *AURKB* | 9212 | NM_004217 |
| *CCNA1* | 8900 | NM_003914 |
| *CCNA2* | 890 | NM_001237 |
| *CCNB1* | 891 | NM_031966 |
| *CCNB2* | 9133 | NM_004701 |
| *CCNB3* | 85417 | NM_033031 |
| *CCNC* | 892 | NM_005190 |
| *CCND1* | 595 | NM_053056 |
| *CCND2* | 894 | NM_001759 |
| *CCND3* | 896 | NM_001760 |
| *CCNE1* | 898 | NM_001238 |
| *CCNE2* | 9134 | NM_057749 |
| *CCNF* | 899 | NM_001761 |
| *CCNG1* | 900 | NM_004060 |
| *CCNG2* | 901 | NM_004354 |
| *CCNH* | 902 | NM_001239 |
| *CCNI* | 10983 | NM_006835 |
| *CCNL1* | 57018 | NM_020307 |
| *CCNT1* | 904 | NM_001240 |
| *CDC16* | 8881 | NM_003903 |
| *CDC2* | 983 | NM_033379 |
| *CDC20* | 991 | NM_001255 |
| *CDC25A* | 993 | NM_001789 |
| *CDC25B* | 994 | NM_004358 |
| *CDC25C* | 995 | NM_001790 |
| *CDC27* | 996 | NM_001256 |
| *CDC34* | 997 | NM_004359 |
| *CDC37* | 11140 | NM_007065 |
| *CDC45L* | 8318 | NM_003504 |
| *CDC6* | 990 | NM_001254 |
| *CDC7* | 8317 | NM_003503 |
| *CDK10* | 8558 | NM_003674 |
| *CDK2* | 1017 | NM_001798 |
| *CDK3* | 1018 | NM_001258 |
| *CDK4* | 1019 | NM_000075 |
| *CDK5* | 1020 | NM_004935 |
| *CDK5R1* | 8851 | NM_003885 |
| *CDK5R2* | 8941 | NM_003936 |
| *CDK6* | 1021 | NM_001259 |
| *CDK7* | 1022 | NM_001799 |
| *CDK8* | 1024 | NM_001260 |
| *CDK9* | 1025 | NM_001261 |
| *CDKL1* | 8814 | NM_004196 |
| *CDKL2* | 8999 | NM_003948 |
| *CDKL5* | 6792 | NM_003159 |
| *CDKN1A* | 1026 | NM_000389 |
| *CDKN1B* | 1027 | NM_004064 |
| *CDKN1C* | 1028 | NM_000076 |
| *CDKN2A* | 1029 | NM_000077 |
| *CDKN2B* | 1030 | NM_004936 |
| *CDKN2C* | 1031 | NM_001262 |
| *CDKN2D* | 1032 | NM_001800 |
| *CDKN3* | 1033 | NM_005192 |
| *CDT1* | 81620 | NM_030928 |
| *CENPA* | 1058 | NM_001809 |
| *CENPB* | 1059 | NM_001810 |
| *CENPC1* | 1060 | NM_001812 |
| *CENPE* | 1062 | NM_001813 |
| *CENPF* | 1063 | NM_016343 |
| *CENPH* | 64946 | NM_022909 |
| *CHEK1* | 1111 | NM_001274 |
| *CHEK2* | 11200 | NM_007194 |
| *CKS1B* | 1163 | NM_001826 |
| *CKS2* | 1164 | NM_001827 |
| *CNK* | 1263 | NM_004073 |
| *CRI1* | 23741 | NM_014335 |
| *E2F1* | 1869 | NM_005225 |
| *E2F2* | 1870 | NM_004091 |
| *E2F3* | 1871 | NM_001949 |
| *E2F4* | 1874 | NM_001950 |
| *E2F5* | 1875 | NM_001951 |
| *E2F6* | 1876 | NM_001952 |
| *FOS* | 2353 | NM_005252 |
| *GAK* | 2580 | NM_005255 |
| *GMNN* | 51053 | NM_015895 |
| *HIPK2* | 28996 | NM_022740 |
| *HUS1* | 3364 | NM_004507 |
| *CCNT2* | 905 | NM_001241 |
| *JUN* | 3725 | NM_002228 |
| *JUNB* | 3726 | NM_002229 |
| *MAD2L1* | 4085 | NM_002358 |
| *MAD2L2* | 10459 | NM_006341 |
| *MCM10* | 55388 | NM_018518 |
| *MCM2* | 4171 | NM_004526 |
| *MCM3* | 4172 | NM_002388 |
| *MCM3AP* | 8888 | NM_003906 |
| *MCM4* | 4173 | NM_005914 |
| *MCM5* | 4174 | NM_006739 |
| *MCM6* | 4175 | NM_005915 |
| *MCM7* | 4176 | NM_005916 |
| *MCM8* | 84515 | NM_032485 |
| *MCMDC1* | 254394 | NM_153255 |
| *MDM2* | 4193 | NM_002392 |
| *MKI67* | 4288 | NM_002417 |
| *MNAT1* | 4331 | NM_002431 |
| *MYC* | 4609 | NM_002467 |
| *ORC1L* | 4998 | NM_004153 |
| *ORC2L* | 4999 | NM_006190 |
| *ORC3L* | 23595 | NM_012381 |
| *ORC4L* | 5000 | NM_002552 |
| *ORC5L* | 5001 | NM_002553 |
| *ORC6L* | 23594 | NM_014321 |
| *PCNA* | 5111 | NM_002592 |
| *PIN1* | 5300 | NM_006221 |
| *PLK* | 5347 | NM_005030 |
| *PTGIR* | 5739 | NM_000960 |
| *PTGIS* | 5740 | NM_000961 |
| *RAD1* | 5810 | NM_002853 |
| *RAD17* | 5884 | NM_133338 |
| *RAD51* | 5888 | NM_002875 |
| *RAD52* | 5893 | NM_002879 |
| *RAD9A* | 5883 | NM_004584 |
| *RB1* | 5925 | NM_000321 |
| *RBBP2* | 5927 | NM_005056 |
| *RBL1* | 5933 | NM_002895 |
| *RBL2* | 5934 | NM_005611 |
| *RBP1* | 5947 | NM_002899 |
| *RPA3* | 6119 | NM_002947 |
| *SKP1A* | 6500 | NM_006930 |
| *SKP2* | 6502 | NM_005983 |
| *SNK* | 10769 | NM_006622 |
| *STK6* | 6790 | NM_003600 |
| *TFDP1* | 7027 | NM_007111 |
| *TFDP2* | 7029 | NM_006286 |
| *TP53* | 7157 | NM_000546 |
| *TP73* | 7161 | NM_005427 |
| *TP73L* | 8626 | NM_003722 |
| *YWHAZ* | 7534 | NM_003406 |

| **Table S4:** RT-qPCR primers | |  |
| --- | --- | --- |
| **Gene** | **Forward primer** | **Reverse primer** |
| *HMBS* | GGCAATGCGGCTGCAA | GGGTACCCACGCGAATCAC |
| *HPRT1* | TGACACTGGCAAAACAATGCA | GGTCCTTTTCACCAGCAAGCT |
| *SDHA* | TGGGAACAAGAGGGCATCTG | CCACCACTGCATCAAATTCATG |
| *YWHAZ* | ACTTTTGGTACATTGTGGCTTCAA | CCGCCAGGACAAACCAGTAT |
| *TBP* | CACGAACCACGGCACTGATT | TTTTCTTGCTGCCAGTCTGGAC |
| *TOP2A* | CCATTGGCTGTGGTATTGTA | TCATTGGCATCATCGAGTTT |
| *CCNE2* | AATTGTTGGCCACCTGTATT | CAGGCAAAGGTGAAGGATTA |
| *TK1* | AACAGCATCTTTCACCAAGA | TTCCACCAACCAGTGAATTT |
| *CCND1* | TATTGCGCTGCTACCGTTGA | CCAATAGCAGCAAACAATGTGAAA |
